# Selections on 625 Injections for Local Anesthesia

**Published:** 1910-04-15

**Authors:** Maurice Polet

**Affiliations:** Dentist in Brussels


### SELECTIONS ON 625 INJECTIONS FOR LOCAL ANESTHESIA.

BY MAURICE POLET, DENTIST IN BRUSSELS.

When I was admitted a member of the Society Belge de Stomatolgoie I decided, for the following reasons, to make some statistical investigations with regard to extra and intra-dental injections. The reputation and the responsibility of the operator on the one hand, and on the other the freedom from pain, to which the patient is entitled, render the choice of an anaesthetic a very important matter, more especially in cases of pulpectomy and extraction. The subject also has given rise to much heated controversy in some quarters, and has always excited great attention in this society. Further an excessive fear of cocaine has induced a number of colleagues to institute a search among the newer remedies, but without obtaining the desired result. Finally, there is the discovery of alypin and novocain, which have been praised so much, and the possibility of increasing the effect of these anesthetics, and at the same time insuring a bloodless operation by the addition of adrenalin. When I began my investigations my confidence in cocaine was, as it still is, very great; on the other hand I had none whatever in alypin. At the dental clinic at Louvain, where Professor van Mosuenck initiated some of us into our specialty, cocaine injections were the rule, and there were practically no accidents. One day we tried some samples of alypin, and we had, among other things, a serious accident, which Professor Mosuenck reported to you about two years ago; the patient had two alarming attacks of syncope following the alypin injections.

However, I took up alypin again, when several colleagues, among them Dr. Laporta, of Lierre, began to employ alypin without any accidents and with good results; and Dr. Gorin, of Brussels, injected as much as 8cc. of alypin for thyroidectomies, and found it better than cocaine. In order to test it fully I gave 315 injections.

Almost all the tissues of the tooth and the jaw can act as recipients for the anesthetic, so that we have six ways of directing the anesthetic towards the nerve fibres, viz., the dentine, the dental nerve, the gum, the periosteum, the bone; and the nerve trunks themselves. Injection into the dentine is difficult and uncertain. The method which aims at reaching the nerve trunks themselves especially the inferior dental nerve, is difficult and not uniform in its results. My injections were made into the bone, the pulp, the periosteum, or the gum.

Although it would be at least as interesting for us to begin with the consideration of the preliminary anesthesia for pulpectomy, I must first deal with my experiments with regard to extractions, and give you the results of 576 injections. Later on I shall discuss fifty injections into the pulp.

Of the numerous anesthetics at my disposal, I chose, among the well-known ones, cocaine, alypin, stovaine, and novocain and among the little known ones tropacocaine, Beta eucain, acoin, anesthesin, nirvanin and adrenalin. In fifty cases I also injected a mixture of equal parts of cocaine, alypin and stovain.

As a rule, my injection was made up of a drop of adrenalin 1—1000, 2 cc. of the anesthetic, and 2 gm. of water. I injected the gum on each side of the tooth and gave two injections between the tooth to be extracted and the two neighboring ones, as deeply as possible, and more intraperiosteal than intragingival. I made notes of the age, sex, tooth, diagnosis, quantity of anesthetic, adrenalin and water injected, the time between the injection and the extraction, the pain, the hemorrhage, and whether the injection was given warm or cold, and, finally, any observations. In estimating the pain and hemorrhage I used the terms, "none," "very slight," "moderate," "severe."

For the purpose of comparing results under more or less equal conditions, and so perhaps finding a point in favor of one or other remedy, I divided the extractions into four groups: (1) roots, (2) teeth the subject of pyorrhea, (3) of periositis, (4) the second, third, and fourth degrees of caries.

One hundred and seventy-seven of the 315 injections of alypin produced complete anesthesia, i. e., 58 per cent. If we add to these seventy cases of very slight pain, we get 80 per cent.

Sixty-seven of 134 injections of cocaine produced complete anesthesia, i. e., 50 per cent., and if we add thirty-five instances of very slight pain, we get 77 per cent.

Twenty-six injections of stovaine gave respectively 33 and 50 per cent.

Fifty injections of a mixture of alypin, cocaine, and stovaine gave 61 and 88 per cent.

Eleven injections of novocain gave 20 and 35 per cent.

Nine injections of tropacocaine gave 0 and 33 per cent.

Six injections of Beta eucaine gave much the same result.

Acoin was worse still, and the injections of nirvanin and anesthesin were painful and useless.

Five injections of a saline injection of adrenalin gave rise to slight pain in each case.

One injection of water given by mistake in a person in whom a cocaine injection for a similar tooth had produced total anesthesia gave a like result, except that the injection was slightly painful.

Six intradiploic injections enabled me to perform four painless extractions and two with slight pain. In five of these cocaine was given, and alypin in the sixth. Perhaps I may be permitted to read the notes of my second intradiploic injection: Boy, aged 13½, 1st M.U.L., inflamed pulp, solution of 2 cc. alypin, a drop of adrenalin, 2 cc. of water. One or two drops injected into the gum between the premolar and molar. Drill introduced obliquely upwards and inwards through the external layer of bone. The injection penetrated in ten seconds, without pain and without loss. Interval of five minutes. After this I found the gum insensitive to a prick on the outer side from the 3d M.U.L. to the left central incisor, whereas on the inner side it was only insensitive as far as the two premolars. The patient noticed the anesthesia as far as the right canine. He felt occlusion, pressure, and percussion much less on the left side than on the right. On extraction there was some slight pain, chiefly on the inner side; the hemorrhage was very slight. The operation lasted ten minutes.

I have never had complaints regarding phenomenas taking place following the operation.

Few patients have sometimes complained of cephalalgia the evening following the injection, but I do not think wise to incriminate the injection as the cause of this phenomena.

The local phenomenas which follow the injection are always immediate—and follow one another—swelling sensation, anesthesia of the mucous membrane, paresis and sometimes paralysis of the muscles of the region. Then the dentine, the pulp and the nerves are anesthetized.

It asepsis has been sufficiently exercised there will be no trouble following. In one instance I noticed a slight case of oedema which lasted for two days; no pain following the injection, and in some cases I was compelled to anesthetize the same tooth three times in five days, and there never was any soreness nor any complaint from the patient of the gum tissue, pericementum or pulp.

This, once more is a proof of the inoffensive action of novocaine on the tissues.

The period of anesthesia varies from one to three hours, according to the dose injected, and a great advantage is that the cases where the removal of the pulp is required, the action of the adrenalin chlorid as an hemostatic is such that two or three cotton points are sufficient to dry the root-canals after the removal of the pulp.

Upper and lower molars are very difficult to anesthetize. The main point in these cases is to locate the direction of one of the roots and the possibility of reaching the apical region with the injection. It must be noticed that I never use more than one injection where the apex is easily accessible.

Of one thousand injections I have made in the past ten months, here are *grosso-modo* the results: Upper teeth, even cuspids, are very readily anesthetized.

The lower teeth are somewhat difficult, especially the cuspids. Lower and upper moloars are usually anesthetized with great difficulty although anesthesia may be obtained if the apical region is reached with the injection.

If there is malposition of the teeth, the anesthesia is more difficult to obtain, although I have always succeeded in such cases.

The advantages of this method are numerous as they enable us to cut rapidly into sensitive dentine without inflicting pain to the patient, thereby allowing us to perform restorations in much less time.

In classifying the extractions into four groups, it is necessary to bear in mind that an injection may be a comparative success in periostitis (especially with adrenalin) and much better in the case of an intact tooth, and best of all in the case of roots.

Cocaine seemed to give the best results in the second, third, and fourth degrees of caries; alypin seemed best for roots. The mixture of alypin, cocaine, and stovaine succeeded very well in periostitis. Needless to say, thousands of injections are necessary to study these details and confirm the results.

A point of great interest is the addition of adrenalin. Dr. Roy advises the addition of 2 to 4 drops of adrenalin, 1—1000 to 1 cc., and after that only half as much.

If we place those cases together in which the *pain was nil*, or very slight, injections without adrenalin compare as follows with those given with adrenalin:—

| | Without Adrenalin. Per cent. | With Adrenalin. Per cent. | Difference. Per Cent. |
|---|---|---|---|
| Alypin | 76 | 91 | 15 |
| Cocaine | 58 | 81 | 23 |
| Stovaine | 40 | 52 | 12 |
| Novocaine | 27 | 32 | 5 |
| Tropacocaine | 27 | 32 | 5 |
| Eucain | 27 | 32 | 5 |

The addition of adrenalin, therefore, improves the anesthetic power of cocaine and alypin by 19 per cent., i.e., from 67 to 86 per cent., almost a third. Adrenalin diminishes the hemorrhage. The latter is *nil*, or very slight, in 70 per cent. of the cases which alypin, in 80 per cent. with cocaine, which has in itself a vaso-constricting effect, in 83 per cent,. with a mixture of cocaine, alypin, stovaine, and in 60 per cent. with stovaine.

One would expect that adrenalin would have some effect in periostitis; it diminishes the size of the arterioles and hinders the escape of the anesthetic into the general circulation. The cases of periostitis treated without adrenalin were painless, or almost so, in 50 per cent.; if treated with adrenalin, in 63 per cent.; and if treated with adrenalin and cocaine, in 68 per cent.; if with a mixture of cocaine, stovain, and alypin, in 83 per cent; and if with alypin in 63 per cent.

It is useful to give the injection warm, and to use a 7 per cent. saline solution.

The time to wait seems to be about five minutes after the last injection.

I saw two cases of syncope and thirteen cases of threatened syncope. Cocain caused syncope twice, and threatened syncope four times. The first case of syncope came on three hours after an injection of 1½ cc. Syncope was threatened by alypin once, by the mixture once, by eucaine once, and by novocaine twice. The two other cases were the result of the prick of the needle.

The quantity necessary for an injection varies according to the tooth. I can obtain complete anesthesia with ½cc. of alypin. The average dose is 1 to 1½ cc. The addition of adrenalin reduces the amount of anesthetic necessary. The relations obtaining between the pain and the hemorrhage are constant, and this proves that the adrenalin, acting on the arterioles and the axis cylinders, reinforces the action of the anesthetic. As adrenalin retards the entrance of the drug into the general circulation, it reduces the cases of threatened syncope—in other words, it ensures a very good anesthetic.

I now give you the results of fifty intrapulpar injections, which were produced either by compression or cocaine-adrenalin injection.

The pulpectomies were quite painless.

Two cases presented some difficulty.

In four cases pain lasting two to twelve hours followed the operation.

In six cases the nerve was killed and the tooth filled with "Trio" paste at one sitting. In one of them the tooth had been painful to pressure for two days.

I once tried alypin for an intrapulpal injection, but the hemorrhage was so great that I gave it up.

I hope to be able to give you some further statistics in a year or two, when I have a larger number of cases. In any case it seems to me that alypin is less dangerous and more efficient than cocaine, and adrenalin is certainly a useful and necessary adjunct.—T. L. Larseneur, in Feby. 1910, *American Journal.*

